# Does the use of proton pump inhibitors increase the risk of hypomagnesemia: An updated systematic review and meta-analysis

**DOI:** 10.1097/MD.0000000000015011

**Published:** 2019-03-15

**Authors:** Shengtao Liao, Li Gan, Zhechuan Mei

**Affiliations:** a Department of Gastroenterology, The Second Affiliated Hospital of Chongqing Medical University, Chongqing; b Department of Anatomy, North Sichuan Medical College, Nanchong, Sichuan, P.R. China.

**Keywords:** hypomagnesemia, meta-analysis, proton pump inhibitors, systematic review

## Abstract

**Background:**

Proton pump inhibitors (PPIs) are commonly used in the treatment of acid-related diseases; however, the association between the use of PPIs and potential risk of hypomagnesemia is controversial.

**Methods:**

In the present study, databases including PubMed, EMBASE, MEDLINE, PsycINFO, CINAHL, the Cochrane Library, and 4 Chinese databases were searched since the inception until April 2018. Previous observational studies on the incidence of hypomagnesemia in individuals exposed to PPIs were included.

**Results:**

This systematic review involved 15 studies including 129,347 participants, and the sample size varied from 52 to 95,205. Meta-analysis of 14 studies indicated that the use of PPIs increased the risk of hypomagnesemia [RR, 1.44, 95% CI, 1.13–1.76; I^2^, 85.2%]. Subgroup analysis revealed that the use of PPI was not associated with the incidence of hypomagnesemia in outpatients [RR, 1.49; 95% CI, 0.83–2.14; I^2^, 41.4%] and hospitalized patients [RR, 1.05; 95% CI, 0.81–1.29; I^2^, 62.1%], respectively. The use of PPIs was not related to the risk of hypomagnesemia based on the cut-off values of 1.8 mg/dL [RR, 1.73; 95% CI, 0.87–2.58; I^2^, 65.2%], 1.7 mg/dL [RR, 1.48; 95% CI, 0.90–2.06; I^2^, 87.6%], and 1.6 mg/dL [RR, 0.98; 95% CI, 0.69–1.27; I^2^, 67.9%].

**Conclusion:**

The association between the exposure to PPI and the incidence of hypomagnesemia remained unclear. Due to the remarkable heterogeneity in previous studies, a definitive conclusion could not be drawn. Further research should be conducted to investigate the relationship between the use of individual PPI and potential risk of hypomagnesemia, and a dose-response analysis may be required.

## 1 Introduction

Proton pump inhibitors (PPIs) are widely used in the treatment of acid-related diseases including gastroesophageal reflux, functional dyspepsia, and peptic ulcer.^[1]^ PPIs are generally effective and well tolerated; however, concerns on the long-term use of PPIs have already been raised, as PPIs may induce some side effects such as acute interstitial nephritis,^[2]^ clostridium difficile colitis,^[3]^ hospital-acquired pneumonia,^[4]^ hip fracture,^[5]^ osteoporosis, drug interaction, micronutrient deficiency, renal disorder, and dementia.^[6,7]^ Recently, severe hypomagnesemia has been reported in patients treated with PPIs. Two systematic reviews have revealed the association between the use of PPIs and potential risk of hypomagnesemia, suggesting that PPIs treatment may increase the incidence of hypomagnesemia;^[8,9]^ however, due to the notable heterogeneity among the studies, no definitive conclusion could be drawn.

In addition, previous observational studies have indicated that the risk of hypomagnesemia is associated with the use of PPIs, but the results were controversial. For example, Chowdhry et al^[10]^ investigated the clinically significant alteration of serum magnesium levels in 2400 patients treated with various PPIs at different dosages, with or without diuretics. The results revealed that mean magnesium levels remained unchanged in patients treated with PPIs (*P*=.40), and there was no statistical difference in the prevalence of hypomagnesemia (14.7% vs 15.1%, *P*=.77). Thus, a systematic review of previous observational studies was conducted by analyzing the available data on the association between the use of PPIs and potential risk of hypomagnesemia.

## 2 Methods

### 2.1 Inclusion and exclusion criteria

#### 2.1.1 Types of studies

Previous observational research such as cohort studies and case-control or cross-sectional studies which evaluate the risk of hypomagnesemia in patients treated with PPIs were included. Odds ratios, relative risks (RR), or hazard ratios (HR) with 95% confidence intervals (CIs) were presented. Ethical approval was not required considering the nature of the study. Studies were excluded if the outcomes of interest were not reported; the effect sizes were not provided or could not be calculated using the data provided.

#### 2.1.2 Types of participants

Patients exposed to PPIs were included. There was no restriction with respect to indications for the use of PPIs.

#### 2.1.3 Types of exposure and comparisons

The exposure of interest was the use of PPIs. Participants without PPIs treatment were used as control.

#### 2.1.4 Types of outcome measurements

The outcomes of interest were the potential risk of hypomagnesemia.

### 2.2 Search strategy

Previous studies were searched on the databases including PubMed, EMBASE, and the Cochrane Library by 2 independent reviewers since inception until April 2018. Additional studies in the reference lists of all the identified publications were referred, such as relevant meta-analyses and systematic reviews. The terms “proton pump,” “dexlansoprazole,” “esomeprazole,” “ilaprazole,” “lansoprazole,” “omeprazole,” “pantoprazole,” “rabeprazole,” “hypomagnesemia,” “hypomagnesaemia,” and “magnesium” were used in the searches.

### 2.3 Selection of studies and data extraction

Two reviewers screened the titles and abstracts of each search record independently. Entire articles were obtained when either the information provided in the title or abstract matches the aforementioned selection criteria, or the inclusion eligibility could not be ascertained due to the limited information provided. For the studies included, relevant data were extracted by each reviewer and entered into a standardized form. Following information were included in the data extraction form: general study characteristics; general patient characteristics; study design; sample size; exposures and comparisons; and outcomes of interest with effect size and 95% CI. Discrepancies were resolved by consensus.

### 2.4 Quality assessment

Two reviewers assessed the methodological quality of identified studies independently. The quality of observational studies was evaluated using the Newcastle–Ottawa quality assessment scale (http://www.ohri.ca/programs/clinical epidemiology/oxford.asp) as recommended by the Cochrane Collaboration.^[11]^ The maximum score on the Newcastle–Ottawa quality assessment scale is 9. In the present review, a score of 7 to 9 was considered as high, 4 to 6 as moderate, and 0 to 3 as low quality, respectively. Disagreements were discussed and agreed upon consensus.^[12]^

### 2.5 Statistical analysis

Meta-analyses were performed by calculating the pooled RRs with 95% CIs. By performing a conservative approach, random effect model that produces wider CIs compared with fixed effect model was used. The *P* values were 2-tailed, and a value of.05 was considered to indicate a statistically significant difference in all tests apart from heterogeneity. Meta-analyses were conducted and data were presented using Stata 12.0 (Stata Corporation, College Station, TX). The subgroup analysis was performed using various settings, cut-off values, and study types. The sensitivity analysis was conducted by excluding the trials with low quality.

## 3 Results

### 3.1 Literature search

**Figure 1:**
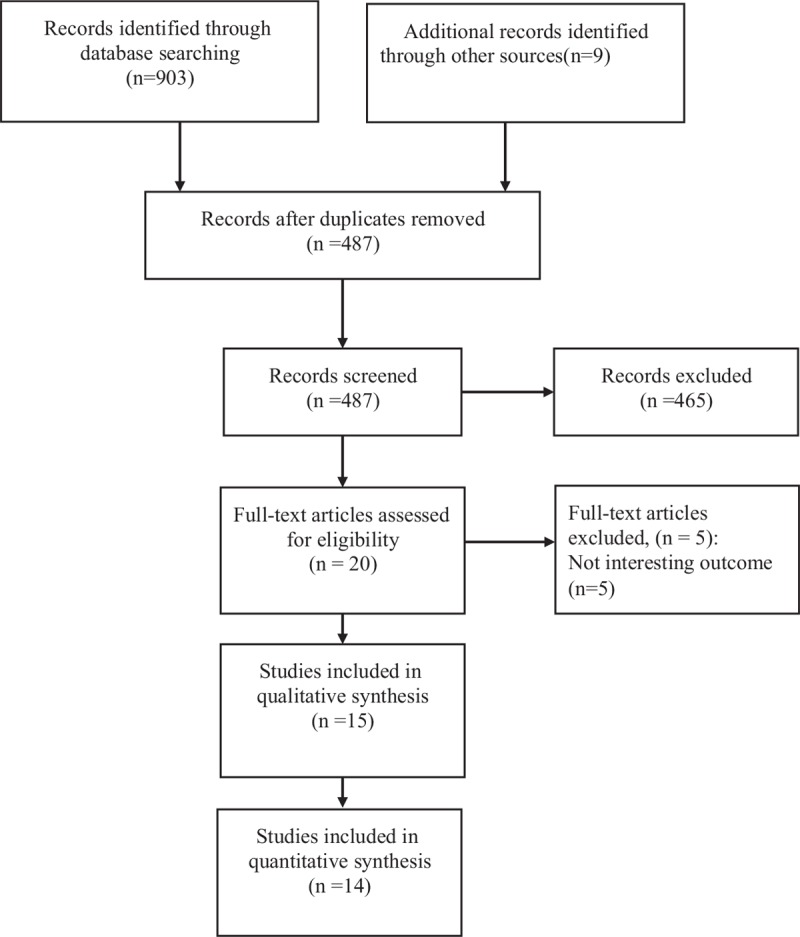
Flowchart of the literature screening.

In the present review, 912 search results were initially identified. Then duplicates were removed, the titles and abstracts were screened and subsequently the entire articles were reviewed. A total of 15 observational studies (n = 129, 347) met the inclusion criteria, including 10 cross-sectional, 1 case-control, and 4 cohort studies (Fig. 1).

### 3.2 Study characteristics

The characteristics of identified studies were presented in Table 1.^[10,13–26]^ A total of 15 studies involving 129,347 participants, with sample sizes varying from 52 to 95,205 were included in the present review. The first authors were from the United States of America (7/15, 46.7%), the Netherland (1/15, 6.7%), Switzerland (1/15, 6.7%), Japan (1/15, 6.7%), Korea (1/15, 6.7%), Belgium (1/15, 6.7%), Brazil (1/15, 6.7%), Croatia (1/15, 6.7%), and Israe (1/15, 6.7%). The age of participants ranged from 18 to 94 years old. Seven studies recruited participants with numerous diseases, including renal transplant recipients (2/15), hematopoietic cell transplant recipients (1/15), patients with late-stage renal diseases on hemodialysis, and acute or chronic kidney disorders treated with hemodialysis (4/15). Ten studies enrolled participants with various settings, such as hospitalized patients in 7 studies, outpatients in 2 studies and inpatient or patients from emergency department in 1 study. Additionally, 12 studies investigated the effects of confounding factors, including age, sex, race, and comorbidities.

**Table 1:**
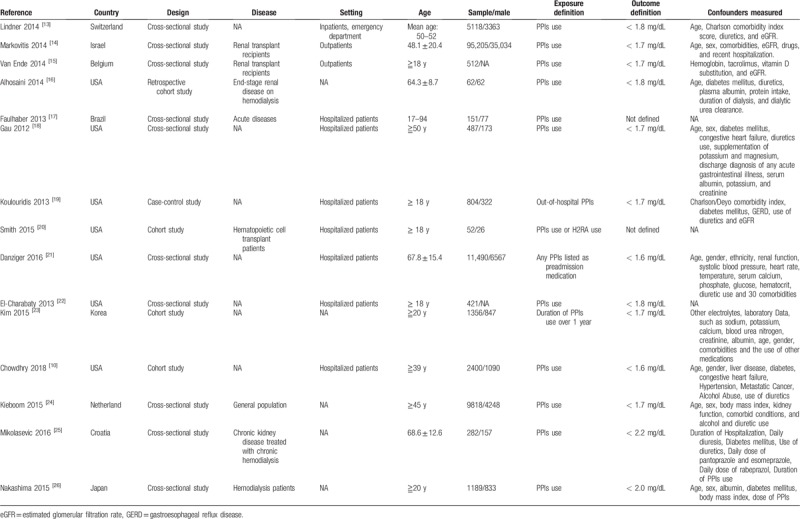
The characteristic of included studies.

### 3.3 Quality assessment

A total of 13 studies (86.7%) were rated as high quality (scoring 7.54 ± 0.66), 2 (2.9%) were rated as moderate (scoring 6), and no study with low quality was included (Table 2). One study was rated with the highest score in the selection outcome, 13 were scored as the highest in the comparability outcome, and 7 were rated with the highest score in the exposure outcome (Table 2).

**Table 2:**
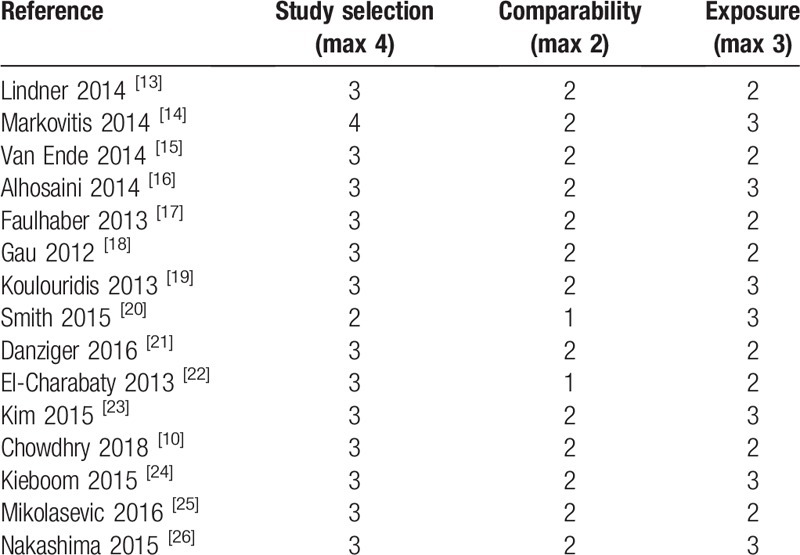
Risk of bias in included studies.

### 3.4 Potential risk of hypomagnesemia in patients treated with PPIs

**Figure 2:**
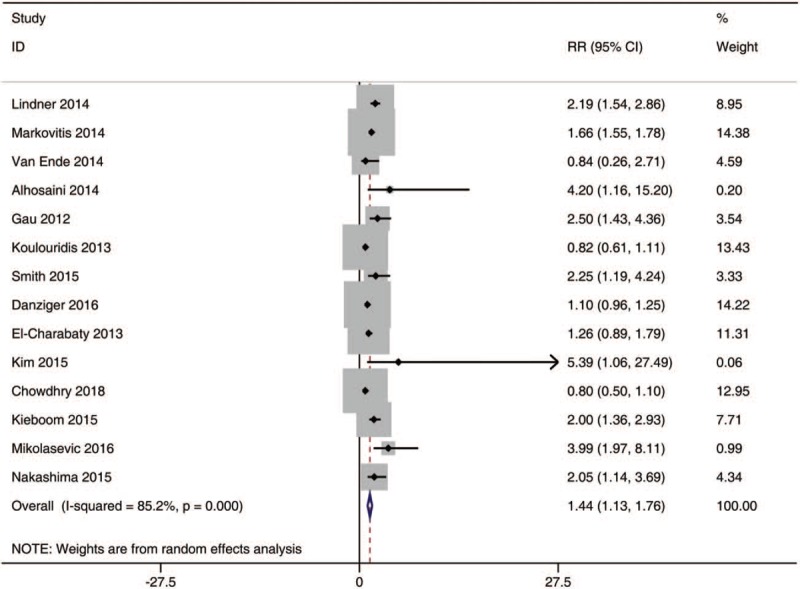
The risk of hypomagnesemia in PPI users. PPI = proton pump inhibitors.

A total of 14 observational studies with 129,347 patients enrolled were used in the data analysis. The pooled RR was 1.44 [95% CI, 1.13 to 1.76; I^2^, 85.2%] within the participants exposed to PPIs compared with those without PPIs treatment (Fig. 2).

## 4 Subgroup analyses

### 4.1 The incidence of hypomagnesemia in patients with various settings

**Figure 3:**
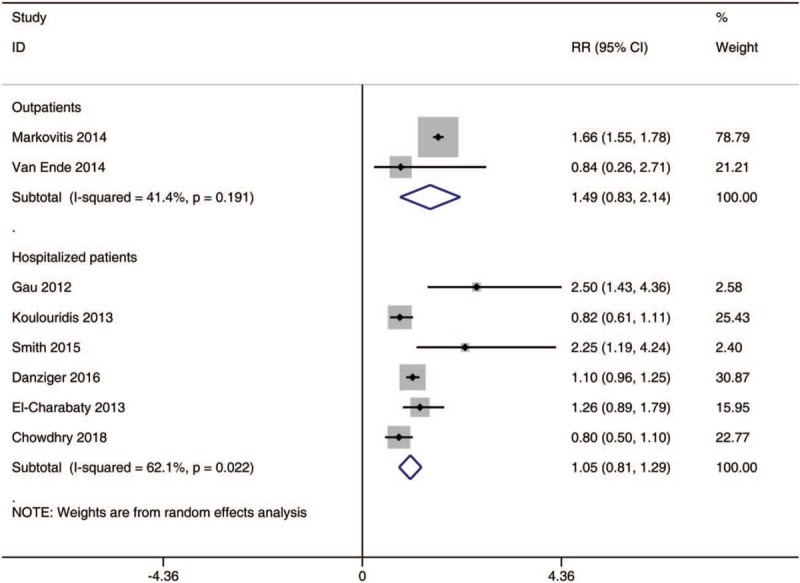
The incidence of hypomagnesemia in PPI users with various setting. PPI = proton pump inhibitors.

In the subgroup analysis on the risk of hypomagnesemia in patients with different setting (Fig. 3), the meta-analysis revealed that the use of PPI was not associated with the incidence of hypomagnesemia in outpatients [RR, 1.49; 95% CI, 0.83–2.14; I^2^, 41.4%] and hospitalized patients [RR, 1.05; 95% CI, 0.81–1.29; I^2^, 62.1%].

### 4.2 The risk of hypomagnesemia with different cut-off values

**Figure 4:**
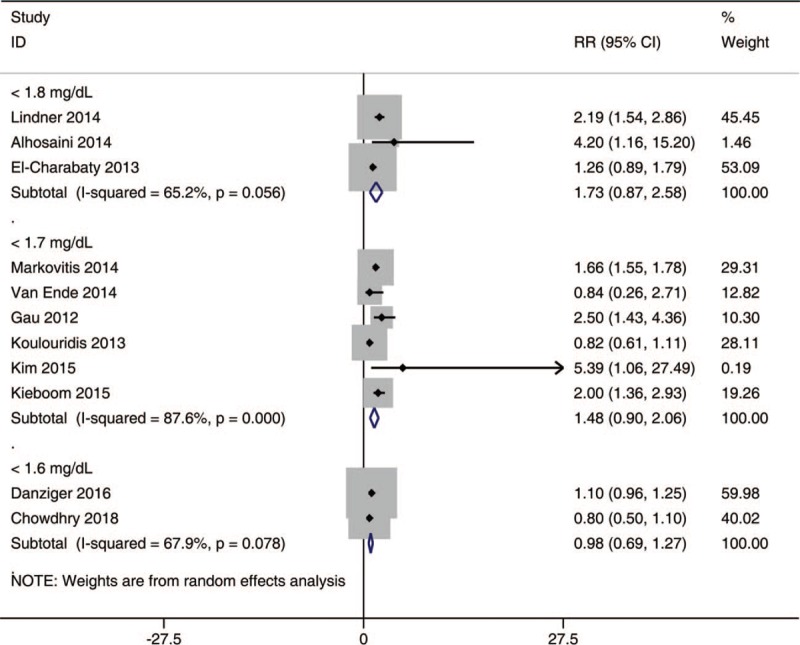
The risk of hypomagnesemia in PPI users at different cut-off values. PPI = proton pump inhibitors.

To investigate the incidence of hypomagnesemia in patients with various cut-off values (Fig. 4), the meta-analysis was performed. The results indicated that there was no association between the use of PPIs and potential risk of developing hypomagnesemia with different cut-off values of 1.8 mg/dL [RR, 1.73; 95% CI, 0.87–2.58; I^2^, 65.2%], 1.7 mg/dL [RR, 1.48; 95% CI, 0.90–2.06; I^2^, 87.6%], and1.6 mg/dL [RR, 0.98; 95% CI, 0.69–1.27; I^2^, 67.9%].

### 4.3 The incidence of hypomagnesemia in various study types

**Figure 5:**
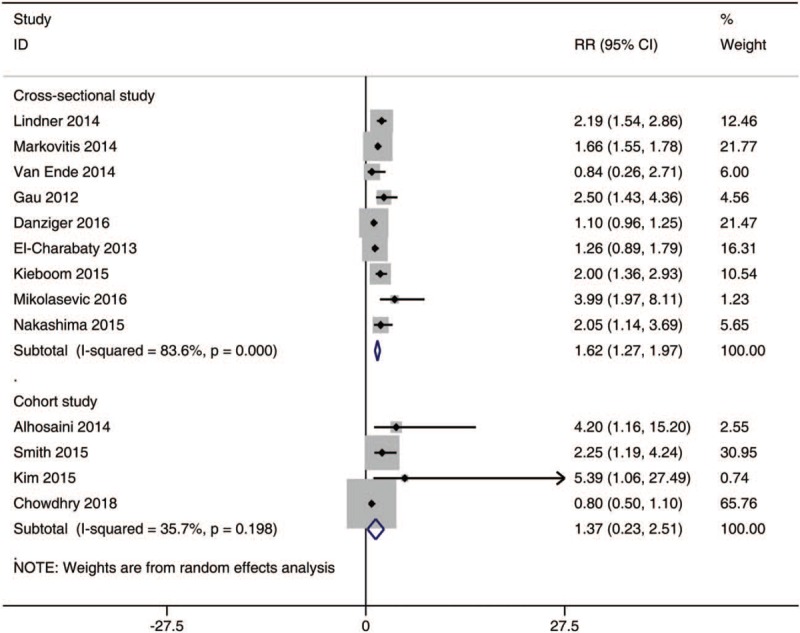
The incidence of hypomagnesemia in PPI users with a variety of study types. [RR, 1.62; 95% CI, 1.27 to 1.97; I^2^, 83.6%]. [RR, 1.37; 95% CI, 0.23 to 2.51; I^2^, 35.7%]. PPI = proton pump inhibitors, CIs = confidence intervals, RR = relative risks.

In the subgroup analysis on the risk of hypomagnesemia in patients with different study types (Fig. 5), the meta-analysis suggested that the use of PPIs was not correlated with the incidence of hypomagnesemia in cross-sectional studies [RR, 1.62; 95% CI, 1.27–1.97; I^2^, 83.6%] and cohort studies [RR, 1.37; 95% CI, 0.23–2.51; I^2^, 35.7%].

### 4.4 Sensitivity analysis

After the trials with low quality were excluded, no material change of the pooled estimated effects was found in sensitivity analysis, and the pooled RR was 1.36 [95% CI, 1.28–1.44; I^2^, 87.3%] in patients exposed to PPIs compared with the ones without PPIs treatment.

## 5 Discussion

In the meta-analyses, existing evidence indicated that PPIs users exhibited ∼1.4-fold increase on the risk of developing hypomagnesemia compared with the control, but remarkable heterogeneity could affect the results of meta-analyses. However, as the subgroup analyses were performed in the present review using different settings, various cut-off values and study types, the effects of heterogeneity were notably reduced. Furthermore, the studies with good quality were selected and used in this systematic review; however, there are still some limitations in the present study: certain important confounding factors such as age, comorbidities, and concomitant medications were not clarified in some studies; there was remarkable heterogeneity among the studies. Although the influences of heterogeneity had been reduced by the subgroup analyses, multiple factors such as different settings, cut-off values, and study types may contribute to the heterogeneity.

Two systematic reviews ^[8,9]^ have indicated the association between the use of PPIs and potential risk of hypomagnesemia, and both studies suggested that exposure to PPIs may increase the incidence of hypomagnesemia, However, previous reviews have only included 9 studies, and significant heterogeneity among the studies does exist. In the present review, increased risk of hypomagnesemia in PPI users was confirmed, and some factors that may contribute to the heterogeneity were also identified. Surprisingly, the increased risk of hypomagnesemia was not found in subgroup analysis, which could be caused by confounding factors such as different settings, cut-off values, and study types. A large amount of studies have been conducted to evaluate the association between the use of PPIs and potential risk of hypomagnesemia, and most research were analyzed in this meta-analysis; however, the outcomes of interest were not identified in some studies. Even if these studies were not included in this review, no association between the use of PPIs and potential risk of hypomagnesemia was found.

A large cohort study was performed by Park et al^[27]^ to examine the levels of serum magnesium in response to PPIs treatment. A total of 2892 patients hospitalized for percutaneous coronary intervention were enrolled in the study, and the results revealed that the incidence of hypomagnesemia (<1.6 mg/dL) was 0.4% (3/834) and 0.4% (1/242) in the patients treated with PPIs and the control group, respectively (*P* = .904).

In addition, Bahtiri et al^[28]^ reported that the levels of serum magnesium remain unchanged in 250 participants after 12 months of PPIs treatment, and no association between the use of PPIs and potential risk of hypomagnesemia was found in healthy donors. Therefore, these findings are also important and should be further reviewed.

Furthermore, variant alleles of transient receptor potential membrane melastatin 6 and 7 (TRPM6/TRPM7) are associated with subtle malabsorption and/or persistent urinary leakage that may be aggravated by PPIs, consequently leading to hypomagnesemia in susceptible individuals. Bai et al^[29]^ revealed that the concentration of [H^+^] could affect the binding of magnesium to TRPM6/TRPM7 during magnesium transport. Thus, the change of pH in intestinal lumen could influence the activity of TRPM6/TRPM7 channels where the binding and absorption of magnesium take place. Therefore, the use of PPIs should be avoided in the treatment of patients with TRPM6/TRPM7 mutation to reduce the incidence of hypermagnesemia.

However, there are some limitations in the present study: previous studies published in English were reviewed, thus some research published in other languages may not be included; certain information such as the type of PPIs and duration of PPIs treatment were not clarified in some studies, thus the effects of individual PPIs could not be investigated and dose-response analysis was not performed to evaluate the influences of different PPIs and treatment duration on hypomagnesemia; this is a meta-analysis of previous observational studies with its inherent limitations. Therefore, further studies need to be designed and conducted to minimize these limitations.

In the present study, remarkable heterogeneity was found when the data from previous studies were pooled. Although potential confounding factors had been adjusted in most studies, some clinical variations in patients such as age, the type of PPIs, the type of diseases, the settings of participants, and comorbidities could still lead to the heterogeneity. Therefore, future research should focus on the influences of PPIs on patients with specific diseases and in certain age group to minimize the clinical heterogeneity. In addition, to evaluate the severity of PPIs-induced hypomagnesemia and identify the high-risk groups, it is necessary to establish a global multicenter registration platform.

The present review revealed that exposure to PPIs may increase the risk of hypomagnesemia. Additionally, some epidemiological studies have indicated the association between hypomagnesemia and the risk of recurrent coronary heart disease and serious arrhythmias.^[30,31]^ Therefore, PPIs derived drugs could be used in the treatment of patients with cardiovascular diseases and hypomagnesemia in clinical practice.

## 6 Conclusions

The association between the exposure to PPIs and potential risk of hypomagnesemia remained unclear. Due to the remarkable heterogeneity in the studies, a definitive conclusion could not be drawn. Further research should be conducted to investigate the relationship between the use of individual PPI and potential risk of hypomagnesemia, and a dose-response analysis may be required.

## Author contributions

ZM and SL designed this research. SL and LG performed the study. SL analyzed the data. All authors have read and approved the final manuscript.

**Conceptualization:** Zhechuan Mei.

**Data curation:** Shengtao Liao, Li Gan, Zhechuan Mei.

**Formal analysis:** Shengtao Liao, Li Gan, Zhechuan Mei.

**Investigation:** Shengtao Liao, Zhechuan Mei.

**Methodology:** Shengtao Liao, Zhechuan Mei.

**Project administration:** Li Gan, Zhechuan Mei.

**Resources:** Li Gan, Zhechuan Mei.

**Software:** Li Gan.

**Validation:** Li Gan.

**Visualization:** Li Gan.

**Writing – Original Draft:** Shengtao Liao, Li Gan, Zhechuan Mei.

**Writing – Review & Editing:** Zhechuan Mei.
